# Guidelines for the Treatment of PTSD Using Clinical EFT (Emotional Freedom Techniques)

**DOI:** 10.3390/healthcare6040146

**Published:** 2018-12-12

**Authors:** Dawson Church, Peta Stapleton, Phil Mollon, David Feinstein, Elizabeth Boath, David Mackay, Rebecca Sims

**Affiliations:** 1 National Institute for Integrative Healthcare, Fulton, CA 20759, USA; dawsonchurch@gmail.com; 2 School of Psychology, Faculty of Society and Design, Bond University, Robina, Gold Coast, QLD 4229, Australia; rsims@bond.edu.au; 3 Institute of Psychoanalysis, London W9 2BT, UK; mollon@clara.net; 4 Private Practice, Ashland, OR 97520, USA; david@innersource.net; 5 Department of Social Work and Social Welfare, School of Health and Social Care, Staffordshire University, Staffordshire ST42DE, UK; E.Boath@staffs.ac.uk; 6 Asociacion Hispana de EFT, Mexico City 72150, Mexico; eftdmp@gmail.com

**Keywords:** emotional freedom techniques (EFT), veteran, post-traumatic stress disorder (PTSD), clinical guidelines

## Abstract

Clinical EFT (Emotional Freedom Techniques) is an evidence-based method that combines acupressure with elements drawn from cognitive and exposure therapies. The approach has been validated in more than 100 clinical trials. Its efficacy for post-traumatic stress disorder (PTSD) has been investigated in a variety of demographic groups including war veterans, victims of sexual violence, the spouses of PTSD sufferers, motor accident survivors, prisoners, hospital patients, adolescents, and survivors of natural and human-caused disasters. Meta-analyses of EFT for anxiety, depression, and PTSD indicate treatment effects that exceed those of both psychopharmacology and conventional psychotherapy. Studies of EFT in the treatment of PTSD show that (a) time frames for successful treatment generally range from four to 10 sessions; (b) group therapy sessions are effective; (c) comorbid conditions such as anxiety and depression improve simultaneously; (d) the risk of adverse events is low; (e) treatment produces physiological as well as psychological improvements; (f) patient gains persist over time; (g) the approach is cost-effective; (h) biomarkers such as stress hormones and genes are regulated; and (i) the method can be adapted to online and telemedicine applications. This paper recommends guidelines for the use of EFT in treating PTSD derived from the literature and a detailed practitioner survey. It has been reviewed by the major institutions providing training or supporting research in the method. The guidelines recommend a stepped-care model, with five treatment sessions for subclinical PTSD, 10 sessions for PTSD, and escalation to intensive psychotherapy or psychopharmacology or both for nonresponsive patients and those with developmental trauma. Group therapy, social support, apps, and online and telemedicine methods also contribute to a successful treatment plan.

## 1. Introduction

More than 70% of adults worldwide and seven million in the United States currently suffer with post-traumatic stress disorder (PTSD); up to thirty percent of people who have been in a war zone will develop the condition; and half will never reach out for professional help [1,2,3]. Worldwide, conditional PTSD risk is elevated after traumas involving violence and prior exposure to some traumas involving violence is associated with generalized vulnerability to subsequent PTSD [2]. The lifetime prevalence of PTSD varies (relevant to social background and country of residence), ranging from 1.3% to 12.2% [3]. Post-traumatic stress disorder develops following exposure to one or more threatening events such as natural disasters, physical or sexual abuse, violent crime, or warfare [2,3]. The average duration of PTSD for people who do not receive treatment is greater than five years; it is three years if they are receiving treatment; and mean symptom duration is considerably longer than previously recognized [2,4].

Both psychological and pharmacological interventions have been developed for treating the condition. Cognitive and exposure therapies have been effective, with symptom reduction being a reliable benefit, but in a review of studies conducted between 1980 and 2015 of cognitive processing therapy and prolonged exposure with military and veteran populations, two-thirds still met the diagnostic criteria for PTSD at the end of treatment [5]. Another study involving 804 clinicians treating 1931 veterans with prolonged exposure was somewhat more encouraging, but still found 46% meeting the diagnostic criteria for PTSD following treatment [6]. Pharmacological interventions such as selective serotonin reuptake inhibitors (SSRIs) have been shown to reduce symptoms or symptom intensity in approximately 60% of PTSD patients, but only 20% to 30% achieve complete remission, and symptoms tend to return when the medication is no longer being administered [7].

More effective therapies are clearly needed, particularly for the millions who still suffer with PTSD after receiving treatment. This paper examines Clinical EFT (Emotional Freedom Techniques) as a potentially highly effective treatment, and it presents PTSD treatment guidelines that were systematically derived from clinical trials and practitioner input.

### 1.1. The Nature and Treatment of PTSD

Post-traumatic stress disorder symptoms include flashbacks, nightmares, intrusive thoughts, severe anxiety, hypervigilance, sleep disturbance, physical aggression, and poor concentration. These symptoms are associated with deterioration in health, relationships, and job performance as well as efforts to avoid thoughts, memories, situations, and individuals associated with the threatening event or events. Although most individuals are exposed to threatening events during their lifetime, the majority do not develop PTSD [8]. 

### 1.2. Prevalence

Up to 30% of individuals experiencing significant trauma will develop PTSDs [9] with the lifetime prevalence varying from 1.3% to 12.2%, and the 1-year prevalence 0.2% to 3.8% [3]. Higher prevalence is found in lower income areas, in conflict and post-conflict zones, and in countries with less access to mental health care [10]. Personal factors that contribute to susceptibility for developing the disorder include the type of the threatening event, age at exposure, gender, and a pre-existing psychological vulnerability [2]. For instance, adults exposed to traumatic events are more likely to develop PTSD if they were traumatized as children. Women are more than twice as likely as men to experience the disorder [11].

### 1.3. Prognosis

A review of longitudinal studies published between 1998 and 2010 found that in populations exposed to trauma, a mean of 28.8% suffering PTSD-like symptoms one month after the trauma, with a wide range, however, of 3.1% to 87.5%. At 12 months, it was 17.0%, with a range of 0.6% to 43.8% [12]. More than a third of those with PTSD never recover [9]. Post-traumatic stress disorder that is “treatment resistant”, with only symptom management being realistic, is widely recognized as a “common clinical problem” ([13], p. 511). A comprehensive review of the duration of PTSD symptomology in individuals who do not receive treatment found that a mean of 51.7% experienced a remission of symptoms within five months following the traumatic event (range was 8% to 89%) and that PTSD tends to remit in only about half of individuals after a period of more than three years [14]. The prognosis for sufferers also deteriorates if diagnosis occurs more than five months following trauma [14]. 

### 1.4. Diagnosis and Assessment

While definitive biological tests are currently unavailable to assist in the diagnosis and assessment of PTSD, checklists and interviews exploring PTSD symptomology have been developed and validated to assist in confirming PTSD diagnoses. The clinician administered PTSD scale for DSM-5 (CAPS-5) is considered the gold standard for PTSD assessment [15]. This is a 30-item interview, useful across a variety of settings, taking approximately 30 to 60 min to administer [15]. Also, commonly used is the self-report PTSD checklist for DSM-5 (PCL-5), which consists of 20 items and can be completed in less than 10 min [15]. 

### 1.5. Complex PTSD

A separate diagnostic category termed “developmental trauma disorder” or “complex PTSD” has been proposed for individuals traumatized as children [16,17]. While the brain is developing, particularly during the first six years after birth, limited coping resources for severe trauma results in dysfunctional emotional learnings that are especially difficult to treat [18]. Insecure attachment at an early age can also result in neurologically hardwired dystopian beliefs about the nature of relationships and of the world [17]. The World Health Organization [19] was the first major health organization to adopt “Complex PTSD” into its diagnostic guidelines. 

### 1.6. Delayed-Onset PTSD

Post-traumatic stress disorder symptoms sometimes do not appear until months after the traumatizing event. While research into delayed-onset PTSD (defined as onset more than six months after the precipitating event) is limited, a review of existing studies found that “delayed onset PTSD in the absence of any prior symptoms was rare, whereas delayed onsets that represented exacerbations or reactivations of prior symptoms accounted on average for 38.2% and 15.3%, respectively, of military and civilian cases of PTSD” [20].

### 1.7. Conventional PTSD Treatments

Biological as well as psychological factors contribute to the development and maintenance of PTSD symptomology, and both pharmacological and psychological interventions have been shown to significantly reduce PTSD symptomology [8]. Each is briefly discussed here. 

### 1.8. Pharmacological

Most of the research into pharmacological treatments for PTSD has focused on selective serotonin reuptake inhibitors (SSRIs), which have been found to produce a broad effect on PTSD symptoms, including reductions in avoidance behavior, hyperarousal, and intrusive re-experiencing of the event [21]. These changes are associated with improved quality of life. Longer duration of SSRI use, for example 36 instead of 12 weeks, has been associated with higher treatment response. Discontinuation of SSRIs is, however, often followed by a relapse of PTSD symptoms [21,22]. While, pharmacological interventions combined with psychotherapy can be efficacious in the treatment of PTSD, many individuals fail to respond to either [23].

### 1.9. Psychological

Exposure-oriented behavioral interventions are frequently used in the treatment of PTSD. They are based on the principle that fear can be both conditioned and extinguished through exposure [8]. Prolonged exposure therapy utilizes protocols consisting of between eight and 15 sessions, administered once or twice weekly, with a session duration of between 60 and 90 min [24]. Prolonged exposure has been shown to lead to clinical gains in 60% of veterans diagnosed with PTSD [6].

Psychological interventions have evolved to incorporate greater emphasis on cognitions, rather than focusing primarily on symptoms and behaviors [25]. One of the most common of these modalities is cognitive processing therapy (CPT), which concentrates on the modification of maladaptive thinking patterns. Typically consisting of 12 sessions, a course of CPT treatment aims to develop strategies for generating more useful or more accurate thinking patterns and utilizes self-written impact statements of the traumatic event [8]. Cognitive processing therapy is markedly superior to waitlist control groups, and in some studies, has been found to be superior to prolonged exposure techniques [26]. 

Trauma-focused cognitive behavior therapy (TF-CBT) is another psychological intervention used to treat emotional and behavioral difficulties associated with trauma, including PTSD [27]. The goal of TF-CBT is to provide psychoeducation and assist the individual in identifying and coping with emotions, thoughts, and behaviors, using techniques such as relaxation, trauma narrative development and processing, and in vivo gradual exposure. Other cognitive-based therapy protocols, and combined exposure and cognitive therapy protocols, also demonstrate promising results in the treatment of PTSD [28]. Despite these advances, between 46% and 66% of patients still meet the diagnostic criteria of PTSD following cognitive or prolonged exposure therapies and research trials indicate clients do not stay in these types of therapies for the required length of time [5,6,23].

Eye movement desensitization and reprocessing (EMDR) is based on Shapiro’s [29] observation that certain techniques using bilateral eye movements, while focused on a traumatic memory, “caused the simultaneous desensitization and cognitive restructuring of memories” (p. 13). Bilateral eye movements as well as other forms of bilateral stimulation seem to reduce the distress associated with the memories, allowing the development of more adaptive thinking patterns [30]. Research concerning the treatment of PTSD suggests that CBT and EMDR are equally effective to, or exceed the effectiveness of, psychopharmacology in the long term [28]. 

### 1.10. PTSD in Veterans

Post-traumatic stress disorder is particularly prevalent and difficult to treat among individuals with war-related PTSD [2]. Even following a course of treatment, up to two-thirds of veterans still meet the diagnostic criteria of PTSD [5]. Adjustment from deployment and reintegration into family and community life are also negatively impacted by the impaired physical, psychological, social, and occupational functioning that are associated with the disorder. Meanwhile, the financial burden of PTSD in veterans is enormous for both the individual and the government [15]. 

Multiple factors influence the likelihood of a service member developing PTSD. These include a younger age at the time of exposure to the traumatic event, belonging to an ethnic minority, lower socioeconomic status, lower military rank, lower educational level, more frequent and longer deployments, prior psychological problems, and lack of social support [31]. 

### 1.11. Clinical EFT

Developed in the early 1990s, Clinical EFT is a psychophysiological intervention which combines psychological methods drawn from conventional therapies with somatic stimulation. The American Psychological Association (APA) has been providing continuing education credits for courses in EFT since 2011. In the U.K., the National Institute for Health and Care Excellence, a governmental authority, has established a category for treating PTSD, “Combined Somatic and Cognitive Therapy”, which includes EFT. While numerous variants of EFT have been put forth, Clinical EFT is distinguished by having been validated in clinical trials that conform to the APA’s Division 12 (Clinical Psychology) standards for empirically supported treatments [32,33]. The research supporting Clinical EFT has utilized a protocol which has remained stable through three editions of the EFT Manual [34]. The Guidelines for treating PTSD developed in this paper are pertinent for treatment approaches that are based primarily on that Manual, whether they are called “EFT”, “Clinical EFT”, or another variation.

## 2. The Clinical EFT Protocol

The cognitive components of Clinical EFT are drawn from CBT and exposure therapy. A typical sequence in the treatment of PTSD might be to have the client vividly recall details of a traumatizing event (exposure) while pairing the memory with emotionally neutral statements (cognitive reframing). To these cognitive and exposure elements, EFT adds the stimulation of a pre-established set of eight acupuncture points (acupoints) by tapping on them with the fingertips, a form of acupressure [34]. For this reason, EFT is often referred to as simply “tapping”, and the EFT Manual [34] outlines the process that has been validated in trials. The typical process involves, for example, a “Setup Statement” that consists of a reference to the traumatic event or related feelings combined with a self-acceptance statement and acupoint tapping. The wording of a typical Setup Statement might be: “Even though I vividly recall the horror of the bomb blast, I deeply and completely accept myself.” During the Setup Statement, the client taps on an acupoint on the side of the hand. For the remainder of the process, they gently tap with two fingers on the eight or more acupoints listed in the manual [34] which include: start of the eyebrow, side of the eye, under the eye, under the nose, under the lips (chin crease), an inch under the collarbone, under the arm, and top of the head. During this time the client says a “Reminder Phrase” (typically a shortened version of the Setup Statement, e.g., one or two words such as “the horror”). To determine the impact of a given procedure, the intensity of the traumatic event is rated by the client using a Subjective Units of Distress (SUD) rating [35], an 11-point Likert scale ranging from 0 (no distress) to 10 (maximum distress). These ratings, which may be taken numerous times during a single session, guide the therapist in determining the next treatment step.

Six comparative studies and a meta-analysis have addressed the question of whether acupoint tapping is an essential ingredient for the favorable outcomes reported following EFT treatments, or whether the cognitive, exposure, and non-specific therapeutic elements of the protocol are the primary active ingredients [36]. Otherwise identical protocols with and without the acupoint tapping component were compared, and those which included tapping produced a significantly larger effect size than those with the other components but without tapping. 

### 2.1. Changes in Biological Markers Following EFT Treatments

As might be expected from a therapy that includes a strong somatic component, measurable changes in biological markers have been found following EFT treatments. Hormonal shifts have been identified, including reductions in cortisol production that were statistically greater than cortisol reductions following a supportive counselling session [37]. Changes in blood flow patterns within the brain have been identified in pre- and post-treatment fMRI readings [38]. Studies examining the epigenetic effects of EFT treatment have found regulation of a range of genes related to health and mental health [37,39,40]. For instance, a one-hour EFT psychotherapy session found that EFT was associated with the regulation of 72 genes, including those involved in learning and memory, regeneration of neural white matter, enhanced synaptic connectivity, neuronal survival after DNA damage, tumor suppression, insulin regulation, heightened immunity, inflammation, and antiviral activity [39].

### 2.2. Efficacy

Evidence demonstrating the effectiveness of EFT and other acupoint psychotherapies has steadily increased in recent years. A systematic review of literature through April 2012 [41] identified 51 outcome studies, including 18 randomized controlled trials (RCTs). Six years later, more than 100 clinical trials, with nearly half being RCTs, have consistently shown EFT to produce positive clinical outcomes (available from the online database maintained at www.Research.EFTuniverse.com). The approach has also been the subject of more than 40 peer-reviewed concept papers and systematic reviews which have established it as an evidence-based treatment for a range of disorders [42]. For instance, meta-analyses of EFT for anxiety [43], depression [44], and PTSD [45] each found large effect sizes (Cohen’s d or Hedge’s g above 0.8).

### 2.3. Treating PTSD with Clinical EFT

The PTSD meta-analysis examined seven RCTs [45]. Emotion Freedom Techniques had a large treatment effect compared to control groups who received standard care, produced equivalent outcomes to other evidence-based therapies—specifically CBT and EMDR—and its effect sizes exceeded those of psychopharmacological interventions. As a result of such encouraging findings, Clinical EFT is finding its way into a variety of traditional institutions in the treatment of PTSD. It has, for instance, been used at Fort Hood, the largest US military base, in the “Warrior Combat Stress Reset Program” which combined relaxation techniques with EFT and EMDR for the remediation of PTSD [46]. This group of studies found that the treatment time frame required to remediate PTSD ranged from 4 to 10 sessions [45].

### 2.4. Populations that Respond

The efficacy of EFT for PTSD has been investigated in a variety of populations, including war veterans, victims of sexual violence, the spouses of PTSD sufferers, motor accident survivors, prisoners, hospital patients, adolescents, and survivors of natural and human-caused disasters [42]. The clinical outcomes demonstrated in the literature appear to be generalizable to a wide variety of populations and settings. Indeed, the clinical trials of PTSD report that less than 10% of clients make little or no progress with EFT and this is reflected in the low dropout rates. In the seven studies reported in the meta-analysis of EFT for PTSD, the mean dropout rate was under 10% [45].

### 2.5. Safety

A core clinical dilemma when treating PTSD is that traumatic memories need to be addressed and processed in order for recovery to occur, but approaching these memories runs the risk of re-traumatization and resulting exacerbation of symptoms [47,48,49]. Moreover, traumatic memories are stored differently from autobiographical memory, being more in the form of sensory-motor reliving of the traumatic experience, and often in an unintegrated mode that is not yet mediated via language [17,50]. Clinical EFT contains inherent procedural elements that are designed to minimize the danger of re-traumatization by approaching the traumatic memory in graduated steps and reducing arousal at each step, making EFT unusually gentle and safe. The approach leads clients into a position from which the traumatic memory can be narrated without emotional distress. A review of studies involving more than one thousand subjects found no “adverse events” to have been reported ([42], p. 650). Based on the extensive research demonstrating Clinical EFT’s efficacy and safety for treating PTSD, the VA has designated it as a “generally safe therapy” [51].

### 2.6. Group Treatment

In addition to individual treatment sessions, group EFT has also been found to be effective [52]. A unique dynamic within EFT group sessions is called “Borrowing Benefits” (Church, 2013b). Borrowing Benefits refers to the way that a group member’s SUD level on a pre-selected personal issue can be lowered by simply watching and tapping along as another group member is guided by the therapist in front of the group. Several studies show that Borrowing Benefits produces significant clinical gains in participants [53,54,55]. For instance, in a study of 218 veterans and their spouses, PTSD symptomology was evaluated before and after a seven-day retreat in which group sessions that utilized Borrowing Benefits were one of the core therapeutic activities [55]. Prior to participating in the retreat, 83% of the veterans were above the PTSD cutoff on a symptom checklist; following the retreat, this had been reduced to 28%. For the spouses, 29% were in the PTSD range prior to the retreat; 4% following the retreat.

### 2.7. Online, App, and Telemedicine Treatment Sessions

In a study of individuals suffering from fibromyalgia, participants were treated entirely online using an EFT protocol [56]. Significant improvements were found in anxiety, depression, and pain. Another study compared EFT delivered via face-to-face sessions with EFT delivered via telephone with a group of veterans reporting clinical symptoms that fall above the PTSD threshold [57]. The in-person sessions produced significantly stronger outcomes (91% symptom reduction to below the PTSD range vs. 67% for the telephone sessions). While a 67% recovery rate might not be optimal, it is superior to recovery rates identified in meta-analyses of psychopharmacology and in-office talk therapy studies. For instance, a comparison of meta-analytic reviews showing large effect sizes in treating PTSD for cognitive therapies (Hedge’s g = 1.63), exposure therapies (1.08), EMDR (1.01), and the medication Topiramate (1.20) [45]. In contrast, six of the seven studies they reviewed of EFT treatments for PTSD exceeded each of these effect sizes, and all seven showed a large effect. In any case, the preliminary evidence from online and telephone applications of EFT indicates that psychological improvement appears to result from such remote interventions. 

Self-guided online programs for applying EFT in the treatment of PTSD are also available. In addition to providing veterans with access to practitioners of Clinical EFT, the web site of the Veterans Stress Project (www.StressProject.org, a program of the National Institute for Integrative Healthcare) hosts a virtual interactive therapy program called Battle Tap [58]. Battle Tap allows veterans to log on and conduct tapping sessions at the time and place of their choosing and with complete anonymity. After entering the site, they describe their concerns, which the software then integrates into a customized Setup Statement. They are then guided via video sessions recorded with other veterans in which they use Borrowing Benefits to reduce PTSD symptoms. Battle Tap is supplemented by a variety of PTSD apps and other resources recommended by APA Division 56 (Trauma Psychology, https://www.apatraumadivision.org/793/veteran-resources.html#apps). The Battle Tap web site will also refer veterans to EFT apps which are currently under development.

### 2.8. Simultaneous Treatment of PTSD and Comorbid Conditions

Post-traumatic stress disorder is typically comorbid with other diagnoses, most often depression, anxiety, substance dependence or abuse, or chronic pain [15]. Emotion Freedom Technique studies examining the relationships among PTSD and co-morbid conditions have found that reductions in PTSD symptoms correlate with reductions in anxiety, depression, and pain [54,59]. When veterans have been successfully treated for PTSD, levels of traumatic brain injury (TBI) symptoms have also been reduced [60,61]. Emotion Freedom Technique group treatments of PTSD using the Borrowing Benefits method also found simultaneous reductions in depression and anxiety [53,54]. 

### 2.9. Prevention and Resilience

Elevated PTSD symptoms, even if subclinical, have been shown to be a risk factor for a later diagnosis of PTSD as well as depression, alcohol abuse, and health problems [62,63]. Emotion Freedom Techniques have been employed as a pre-emptive measure to treat symptoms early, enhance resiliency, and arrest the progression of the diagnosis. In one study, 21 veterans with elevated but subclinical scores on a standardized PTSD measure were no longer in the elevated range after six EFT sessions [62]. Participant gains were durable, with non-elevated scores maintained at follow-up periods of three and six months.

### 2.10. Summary of Benefits

Existing RCTs and meta-analyses show that the treatment of PTSD with Clinical EFT is associated with extensive health and mental health benefits [37,40,43,44,45,61,63,64,65]. These include: (a) the remediation of clinical PTSD symptom levels in between 84% and 90% of veterans; (b) durable long-term treatment results; (c) generalizability across a variety of populations; (d) relatively short timeframes, generally ranging between 4 and 10 sessions; (e) positive clinical outcomes in group as well as individual treatment settings; (f) efficacy when administered by telephone or online webcams; (g) effectiveness whether offered by life coaches or licensed mental health professionals; (h) improvement in comorbid conditions such as anxiety and depression; (i) cost-effectiveness when compared to conventional treatments; and (j) a minimal risk of adverse events. In terms of physiological impact and benefits, EFT has been shown to (a) reduce levels of stress hormones such as cortisol; (b) diminish symptoms such as pain and traumatic brain injury; (c) produce an epigenetic effect on the expression of stress genes; and (d) improve general markers of health such as resting heart rate, blood pressure, and immunoglobulin.

## 3. Clinical Guidelines for Treating PTSD with EFT

A preliminary set of guidelines for treating PTSD with Clinical EFT was derived from a detailed practitioner survey, a review of empirical evidence, and a study of case histories [58]. The survey was distributed by professional groups whose members utilize EFT in the U.S. and the U.K., as well as via practitioner networks. The survey obtained information about topics such as length of EFT treatment for various PTSD severity levels, PTSD assessment instruments most commonly used, treatment outcomes, and the specific EFT techniques considered most beneficial in treating PTSD. Responses were received from 448 practitioners. Representatives from each of the major professional organizations involved with training in and/or research into EFT were then consulted to refine and finalize the Clinical Guidelines presented here [66]. These organizations included, in the U.S., alphabetically, the Association for Comprehensive Energy Psychology, the Energy Medicine Institute, the Energy Psychology Group, and the National Institute for Integrative Healthcare. In Canada, the Canadian Association for Integrative and Energy Therapies. In the U.K., EFT International (previously known as the Association for the Advancement of Meridian Energy Therapies). In Australia, the Evidence-Based EFT Training Group. In Spanish-speaking countries, the Asociacion Hispana de EFT (AHEFT). In French-speaking countries, the Institut Francais de Psychologique Energetique Clinique (IFPEC).

### 3.1. A Stepped-Care Model

The recommendations in the Clinical Guidelines are based on the “stepped-care” model utilized by both the National Institute for Clinical Excellence in the U.K. (NICE) and the Veteran’s Administration (VA) in the U.S. This model recommends offering the least intrusive and potentially most effective intervention for PTSD treatment first. If a patient fails to benefit from this first step, the next and potentially more intrusive treatment is recommended. Only if these initial steps fail are more intrusive methods such as psychopharmacology and intensive psychotherapy advocated. Integrated care, which considers all aspects of the individual’s wellbeing, and all treatment options, should be maintained throughout the intervention process.

### 3.2. The NICE Guidelines

The NICE Guidelines for the treatment of PTSD suggest identification, assessment, psychoeducation, active monitoring, and referral for further assessment as the first “step” in the treatment of PTSD [67]. Assessments routinely involve taking a psychiatric history and inquiry into co-morbid conditions, addiction, alcohol dependence, physical health conditions, availability of social and family support, housing and other material circumstances. The information gathered may dictate referral to additional agencies. Note that the NICE Guidelines are intended for use in the U.K.’s National Health Service, where the availability of a range of somewhat integrated services is assumed. The second step in PTSD treatment recommended by the NICE Guidelines is psychotherapy, using TF-CBT or EMDR. For individuals who do not adequately respond to the first two steps, the NICE guidelines recommend drug treatment as the third step. 

### 3.3. The EFT Guidelines

The Clinical Guidelines presented here are for the use of Clinical EFT as the selected psychotherapy when treating PTSD. The treatment protocol includes five sessions of Clinical EFT for patients who are at risk as evidenced by subclinical PTSD scores and 10 sessions for patients above the clinical cutoff. The recommendation of 10 sessions is the most conservative in the range identified for effective treatment (i.e., the maximum number of sessions usually required for remediation) in the meta-analysis of EFT for PTSD [46]. These sessions are in addition to treatment as usual. Acupoint tapping can be safely used by clients on a self-help basis, and it is generally taught for managing elevated emotions between sessions. The Guidelines also recommend that clients be offered supplementary resources, including access to online services such as Battle Tap and other tutorials as well as access to social support such as Borrowing Benefits groups that include spouses and other family members. 

A third step in treatment intensity is applied with individuals who still suffer with PTSD symptoms following the first two steps. The third step may also be immediately applied for individuals with PTSD combined with a history of developmental trauma. It includes intensive psychotherapy (greater frequency, longer duration, additional clinical protocols, and/or in-patient treatment if dictated by suicidal ideation or other clinical considerations) or psychopharmacology or a combination of the two. The Clinical Guidelines are summarized in Table 1.

### 3.4. Assessment Method

The Clinical Guidelines for treating PTSD with EFT are based on the PCL-4 (PTSD Checklist-4), a 17-item inventory that is available in military (PCL-M), civilian (PCL-C), and specific trauma (PCL-S) versions [68]. The PCL-4 is highly validated for estimating the subjective severity of an individual’s experience of PTSD symptomology. While a newer version, the PCL-5, has been introduced [69], most of the research published in the PTSD literature was performed using the PCL-4. A PCL-4 score of 35–49 is indicative of PTSD risk in veterans, with a score above 50 being the cut-off for a PTSD diagnosis, and a 10 to 20-point reduction in PCL-4 scores being indicative of clinically significant improvement [70]. The PCL can be administered immediately before or after each session to monitor changes in symptomology. The use of the PCL-4 in the Guidelines is to indicate symptom severity and does not preclude the use of other instruments or the PCL-5 when more research on that version is available.

### 3.5. Applying the Guidelines

The representatives of the major organizations involved with EFT who helped formulate and refine these Guidelines recommended several additional considerations and cautions for applying them. 

### 3.6. Practitioner Discretion

In clinical trials, practitioners are required to deliver a method as described in a written treatment manual, and fidelity to that manual is typically monitored by investigators. This strict adherence to a manualized method is a customary standard in research since it ensures that the method, as tested in one study, is the same method tested in another. However, such formulaic applications of a technique are unusual in clinical practice, especially with experienced practitioners. Clinicians are oriented toward patient well-being and will change and combine methods based on their perceptions of what will work best for the client [7]. Most innovations in both psychology and medicine are due to curious practitioners who tried something new in a challenging client situation [33]. In most settings, clinicians have wide latitude over the type of treatment employed, provided it is evidence-based. While the Guidelines are consistent with the published literature and expert opinion, they are only guidelines. Guidelines are useful to determine a probable course of treatment, but in practice they may be and should be modified by the clinician—client by client and session by session.

### 3.7. Developmental Trauma

While clinical trials have brought symptoms to below PTSD thresholds with 86% to 90% of veterans treated with six sessions of Clinical EFT, there are often a small number of participants who do not respond. It has been hypothesized that the presence of developmental trauma, which was not factored into the research designs, may have been a contributing factor for the non-responders [51]. In developmental trauma, traumatic events occur while the child’s brain, personality, attachment patterns, schemas, and internal models of relationships were still forming. As a result, the neural circuitry of PTSD was firing and wiring in the emotional midbrain during a crucial developmental stage, embedding patterns of stress into the child’s interpersonal neurobiology and attachment templates [18,71]. This type of neurologically formative traumatic experience is believed to shape neural circuitry to a degree that is fundamental to subsequent development [17]. The success of EFT in treating PTSD suggests that it could also be effective with complex personality and relational difficulties that are rooted in childhood trauma [49,71,72]. Clinical EFT includes nonverbal somatic procedures, and since developmental trauma is coded non-verbally and may even occur before the acquisition of language, these are likely to be more helpful than primarily cognitive methods. During initial assessment, developmental trauma in clients presenting with clinical PTSD should be noted as a possible factor which might require the treatment to be oriented around the third step specified in the Guidelines. 

### 3.8. Unqualified Practitioners

Because the basic EFT protocol is relatively easy to learn, uncertified, unqualified practitioners have occasionally used it without preliminary assessment of possible psychiatric diagnoses, leading to unfortunate outcomes. While EFT has been successfully applied in the treatment of PTSD by allied care providers such as qualified life coaches [73], mastery in the topics covered in existing EFT practitioner training programs is essential for responsible application.

### 3.9. Practitioner Training

Of Clinical EFT’s 48 distinct techniques, several have been carefully developed for use in cases of PTSD and developmental trauma [34]. The primary organizations certifying practitioners in EFT require a thorough curriculum-based educational program prior to certification. This academic and practice-based training is oriented toward instilling a deep understanding of the essential principles of EFT and developing a core set of skills for its application. These principles and skills are integrated with other pertinent areas of psychological, psychotherapeutic, and neurobiological knowledge. Practice sessions are evaluated and guidance is provided. Ethical issues that can arise in the practice of EFT are explored. If the training is oriented toward individuals who are not already licensed mental health professionals, additional components include recognition of serious psychiatric disorders, the appropriate use of referrals, and other scope of practice issues. In addition, membership in one of the EFT professional organizations, with their detailed codes of ethics, is strongly advised to program graduates.

### 3.10. Relationship with a Primary Care Physician

Because of the pervasive nature of PTSD symptoms, open communication between the EFT therapist and the client’s primary care physician is strongly recommended. Release forms should be signed early when treating PTSD. 

### 3.11. Self-Help Applications

With millions of people having downloaded EFT self-help guides or participated in online trainings over the past quarter-century, the consensus among those teaching people to use EFT on this basis is that the approach is safe and consistently reduces emotional distress [42]. Practitioners routinely teach EFT to clients early in the therapy and recommend that they use it whenever distressing everyday events occur. 

## 4. Conclusions

Unless cost-effective therapies are implemented rapidly, the estimated cost to the US economy of PTSD in veterans alone could exceed a trillion dollars [63]. If widely adapted, Clinical EFT promises to be such a cost-effective, evidence-based treatment.

Combining acupressure with elements derived from cognitive and exposure therapies, EFT has been validated in more than 100 clinical trials and has demonstrated efficacy for a range of populations and psychological conditions. Treatment effects have equaled or exceeded those obtained from pharmacological interventions and conventional psychotherapy. 

Clinical EFT is generally effective with PTSD in four to 10 sessions. After completion of a high quality yet relatively succinct certification program, it can be responsibly applied in individual or group settings by licensed psychotherapists, allied health care professionals, or qualified life coaches. Simultaneous improvement in comorbid conditions typically accompanies EFT treatments of PTSD.

Clinical Guidelines for applying EFT in the treatment of PTSD have been developed, utilizing a stepped-care approach. Five EFT sessions are recommended for subclinical PTSD symptomology, 10 sessions for clinical PTSD, and intensive psychotherapy or psychopharmacology or both for non-responsive patients or those with a history of developmental trauma. In addition, family support, group EFT utilizing the Borrowing Benefits approach, and online resources may be recommended as adjuncts to individual sessions. As an empirically validated, cost-effective therapy, EFT is recommended as a first-line intervention for clinicians treating PTSD.

## Figures and Tables

**Table 1:** Clinical Guidelines for treating post-traumatic stress disorder (PTSD) with Emotion Freedom Techniques (EFT).

Indicator	Proposed Treatment
PCL-4 * Score of 35–49 (at-risk but subclinical)	1st Step: Treatment as usual plus five individual EFT therapy sessions; one instructional consultation on utilizing the Battle Tap interactive online coach; Borrowing Benefits group therapy sessions, inviting the spouse or other family members, may be recommended.
PCL-4 Score above 50 (PTSD range)	2nd Step: Treatment as usual plus 10 individual EFT therapy sessions; two instructional consultations on utilizing the Battle Tap interactive online coach; Borrowing Benefits group therapy sessions, inviting the spouse or other family members, may be recommended.
PCL-4 Score above 50 with history of developmental trauma or persistence of PTSD symptoms after Steps 1 and 2.	3rd Step: As above, with greater frequency, longer duration, additional clinical protocols, and/or in-patient treatment if dictated by suicidal ideation or other clinical considerations OR psychopharmacology OR both.

* PTSD Checklist-4.
